# Evaluation of AQP4/TRPV4 Channel Co-expression, Microvessel Density, and its Association with Peritumoral Brain Edema in Intracranial Meningiomas

**DOI:** 10.1007/s12031-021-01801-1

**Published:** 2021-02-04

**Authors:** Konstantinos Faropoulos, Afroditi Polia, Chrisi Tsakona, Eleanna Pitaraki, Athanasia Moutafidi, George Gatzounis, Martha Assimakopoulou

**Affiliations:** 1 Department of Neurosurgery, University Hospital of Patras, 26504 Rion Patras, Greece; 2 Department of Anatomy, Histology and Embryology, School of Medicine, University of Patras, 26504 Rion Patras, Greece

**Keywords:** AQP4, TRPV4, Meningiomas, Microvessel density, Peritumoral brain edema

## Abstract

Apart from VEGF-A pathway activation, the existence of peritumoral edema (PTBE) in meningiomas has been correlated with the expression levels of water transporter aquaporin 4 (AQP4). A novel cooperation of AQP4 with the transient receptor potential isoform 4 (TRPV4), a polymodal swelling-sensitive cation channel, has been proposed for regulating cell volume in glial cells. We investigated AQP4/TRPV4 channel co-expression in meningiomas along with the neovascularization of tumors and associate with PTBE. Immunohistochemical staining for AQP4 and TRPV4 expression was quantitatively analyzed in semi-serial sections of archival tissue from 174 patients. Microvessel density was expressed as microvessel count (MVC). PTBE was measured and edema index (EI) was assessed in 23 patients, based on magnetic resonance images (MRI) whereas mRNA levels of AQP4 and TRPV4 were evaluated in these patients using quantitative real-time PCR. High AQP4 was associated with lower–tumor grade (*p* < 0.05). AQP4 and TRPV4 were correlated in benign (WHO, grade I) (*p* < 0.0001) but not in high-grade (WHO, grades II and III) meningiomas (*p* > 0.05). AQP4/TRPV4 levels were independent of EI and MVC (*p* > 0.05). In contrast, EI was correlated to MVC (*p* = 0.02). AQP4/TRPV4 co-expression was detected in both edematous and non-edematous meningiomas. However, most of tumors with larger edema (EI ≥ 2) demonstrated increased levels of AQP4 and TRPV4. Importantly, peri-meningioma tissue of edematous meningiomas demonstrated significantly increased expression for AQP4 (*p* = 0.007) but not for TRPV4 (*p* > 0.05) compared with the main tumor. AQP4 and TRPV4 expression is rather associated with a response to vasogenic edema of meningiomas than with edema formation.

## Introduction

Meningiomas are a common slow growing type of primary intracranial tumor and are subdivided into many histopathological types which are mostly benign (Louis et al. 2016; Mawrin and Perry 2010). Even though these tumors can be found incidentally during an autopsy, they can also cause symptoms depending on their size and the pressure they pose to their adjacent structures. Many of these symptoms are induced by the peritumoral brain edema (PTBE) which occurs frequently (Berhouma et al. 2017). PTBE is a well-known negative prognostic factor as it can exacerbate preoperative neurological deficits, increase the risk of intra-operative complications, and prolong hospitalization time; however, its pathophysiology is yet unclear. Factors like tumor size and histological type, location, and gender have been proposed to relate with PTBE, even in cases of small, peripheral, low malignancy meningiomas (Lee et al. 2008; Pereira-Filho 2010). The implication of E-cadherin, beta catenin (Rutkowski et al. 2018), and hypoxia inducible factor-1 (HIF-1) (Reszec et al. 2013) along with the vascular endothelial growth factor A (VEGF-A) pathway activation and increased vascular supply (Nassehi et al. 2011; Nassehi et al. 2013; Sakuma et al. 2008; Schmid et al. 2010) support the theory that PTBE in meningiomas is vasogenic, e.g., a growing meningioma, results in a further increase of intratumoral venous pressure leading to tumor congestion and accumulation of angiogenic substances, increased permeability of cerebral-pial capillaries and expansion of the PTBE (Hou et al. 2013; Nassehi 2013). Wang et al. (2011) have reported a co-expression of VEGF and AQP4, a plasma membrane water-transporting protein of aquaporin (AQP) family, in tumor cells of supratentorial meningiomas with PTBE. Other studies imply a role for AQP4 in edematous meningiomas independently of grade (Gawlitza et al. 2017; Ng et al. 2009). Moreover, distribution of AQP4 in meningothelial cells has been demonstrated (Mobasheri et al. 2007; Zeleny et al. 2017) and contribution of these water channels to CSF flux across the meninges into the glymphatic system has been proposed (Zeleny et al. 2017).

AQP4 plays a key role in fluid homeostasis of the brain, and it has been associated with many cases of brain edema as such that occurs with malignant tumors, metastasis, or trauma (Filippidis et al. 2016; Papadopoulos and Verkman 2013; Zelenina 2010). Interestingly, AQP4 upregulation in gliomas versus normal brain tissues has been mainly associated with glioma-associated blood-brain barrier disturbance and PTBE (Ikota et al. 2006; Saadoun et al. 2002; Warth et al. 2007; Zhao et al. 2012). Previous evidence reveals a significantly positive relationship of AQP4 upregulation with increased expression of VEGF and HIF-1α proteins, which are involved in neovascularization (Mou et al. 2010; Yang et al. 2012), contrary to occludin expression, a tight junction protein, which is downregulated in brain tumors and meningiomas with PTBE (Park et al. 2006). In this context, it is the leaky vascular bed that is responsible for vasogenic edema formation in brain tumors where AQP4 channels are recruited to maintain cerebral water balance (Lin 2013; Maugeri et al. 2016). Along with its primary function of facilitating trans-epithelial water fluid transport in response to osmotic gradients, AQP4 has also been implicated in various processes like cell migration and neural signal transduction (Verkman 2005).

The role of AQP4 in astrocyte volume regulation has recently been established (Lisjak et al. 2017). In this process, interactions between AQP4 and the transient receptor potential isoform 4 (TRPV4), a polymodal swelling-sensitive cation channel in astrocytic plasma membranes in situ and in primary cultures as well as in transfected cell lines, have been reported (Benfenati et al. 2011). TRPV4 was originally identified as a channel that could be gated by changes in osmolarity, but it also elicits responses to a variety of endogenous and exogenous agonists (Vriens et al. 2004). In retinal Müller glial cells under hypo-osmotic stress, water influx through AQP4 leads to activation of TRPV4 via stretch-sensitive phospholipase A2 and eicosanoid messengers, which results in subsequent increase of Ca^2+^ entry and cell swelling (Iuso and Križaj 2016; Jo et al. 2015). Additionally, Kitchen et al. (2020) have investigated, in vivo, the contribution of membrane translocation of AQP4 to CNS edema formation. The authors present that osmotic dysregulation activates mechanosensitive TRPV4 channel which facilitates an influx of Ca^2+^ ions into astrocytes, promoting AQP4 to relocalize to the plasma membrane through co-operation of two proteins namely protein kinase A (PKA) and calmodulin (CaM). TRPV4 channel expression has been detected in meningiomas (Goutsou et al. 2019). The aim of this study was to investigate the co-expression of AQP4/TRPV4 in meningothelial cells and associate it with PTBE in meningiomas. Furthermore, microvascular density was evaluated and the relationship between AQP4 and/or TRPV4 expression with microvessel density and edema extent was studied. The results were associated with patients’ clinicopathological characteristics and survival.

## Materials and Methods

### Demographic Data and Neuropathology

A total of 174 patients with meningiomas who underwent surgery at our Neurosurgery Department, during a 12-year period, were included in this study. Normal human brain tissue was obtained postmortem (two males, 56 and 28 years). The tissue material in this study is based on additional analyses of the samples from our previous study (Goutsou et al. 2019). The tissue material was evaluated by routine methods for histopathology, including immunohistochemical staining for CD31 to highlight microvessel endothelial cells and Ki-67 index as proliferation marker, and graded according to the diagnostic criteria of the WHO classification system (Louis et al. 2016) (Table 1). Histological types included meningothelial (*n* = 93), fibrous (*n* = 18), psammomatous (*n* = 10), transitional (*n* = 10), microcystic (*n* = 3), secretory (*n* = 3), angiomatous (*n* = 1), atypical (*n* = 30), clear cell (*n* = 1), chordoid (*n* = 2), and anaplastic (*n* = 3). Τhe study population was of Greek (*n* = 170) and non-Greek (*n* = 4) origin. Follow-up for 102 patients was evaluated as the number of months from the date of the diagnostic surgical procedure to that of death or date of last follow-up (April 2020). The median follow-up period was 63 months (range 12–288 months). The use of archival material was in accordance with the University Ethics Commission. This study was considered of minimal risk to patients. All patients were informed and consent. All ethical guidelines and rules were followed to protect patient privacy.

**Table 1:** Main clinicopathological features of the patients, labeling index (LI) of AQP4 and TRPV4 channels, and MVC in tumor specimens

Clinicopathological features	Number	AQP4 LIs Mean ± SD, % (range)	TRPV4 LIs Mean ± SD, % (range)	MVC Mean ± SD (range)
Diagnosis Benign (WHO, Grade I) High grade (WHO, Grade II, and III)	138 36	22.65 ± 31.57^a,c^ (0–95) 11.19 ± 25.32^b^ (0–85)	30.34 ± 31.22^d^ (0–95) 44.80 ± 32.98 (0–90)	38.90 ± 43.06 (3–200) 47.23 ± 49.52 (8–200)
EI (range; 1–9.90) EI = 1 EI > 1 EI < 2 EI ≥ 2	3 20 14 9	63.33 ± 50.57 60.80 ± 33.75 53.28 ± 36.34 73.33 ± 30.41	63.33 ± 33.29 60.29 ± 27.35 62.91 ± 25.53 57.50 ± 31.39	36.33 ± 50.00 42.88 ± 44.85 25.15 ± 26.84^e^ 69.25 ± 54.81
Edematous meningiomas Peri-tumor tissue Main tumor	8 12	80.62 ± 19.89^f^ 41.33 ± 32.57	54.28 ± 30.60^g^ 62.91 ± 25.75	
Age (range, years; 21–86***)*** < 60 ≥ 60	79 95	23.60 ± 33.69 17.15 ± 27.69	29.30 ± 30.26 34.04 ± 33.02	30.01 ± 34.72^h^ 49.16 ± 46.58
Gender Male Female	57 117	18.60 ± 28.67 21.30 ± 31.70	31.78 ± 32.09 33.67 ± 32.03	48.07 ± 53.21 35.79 ± 37.57

The (non-parametric) Wilcoxon’s rank-sum test was performed and the level of significant was defined as *p* < 0.05

*LI* the percentage of positive-labeled cells from the total number of tumor cells counted, *Mean* mean labeling index, *SD* standard deviation

^a^*p* < 0.0001 vs. TRPV4 expression in benign meningiomas

^b^*p* < 0.0001 vs. TRPV4 expression in high-grade meningiomas

^c^*p* = 0.02 vs. AQP4 in high-grade meningiomas

^d^*p* = 0.01 vs. TRPV4 in high-grade meningiomas

^e^*p* = 0.01 vs. MVC in meningiomas with EI ≥ 2

^f^*p* = 0.007 vs. AQP4 expression in main tumor

^g^*p* = 0.3 vs. TRPV4 expression in main tumor

^h^*p* = 0.009 vs. MVC in group of patients ≥ 60 years old

## Measurement of Tumor and Edema Volume and Determination of Edema Index

PTBE was measured in 23 patients, who were operated by the senior neurosurgeon of the research protocol (GG) at the last 5 years (20, WHO grade I, and 3 WHO grade II; 9, male, and 14, female; age range, 39–77 years, mean age, 59.4 years), using 5-mm-thick MRI sections in a 1.5-T scanner. PTBE was found in most of the cases to be irregular in shape and orientation; thus, its extension was determined by the volume of high magnetic signal intensity area around the tumor and the tumor itself in T2-weighted images, minus the volume of the tumor, calculated using post contrast infusion T1-weighted images. For all volume estimations, the ellipsoid formula *V* = 4/3·π·*abc* was used, where *a*, *b*, and *c* are the maximal caudo-cranial anterio-posterior and latero-lateral diameters. For each diameter, two measurements were taken using axial, coronal, and sagittal sections, and the mean value of them was utilized as the maximal diameter. The edema index (EI) as a measure of the relationship between tumor and edema was calculated according to the formula EI = (*V*_tumor_ + edema)/(*V*_tumor_). Tumors without PTBE had an edema index of 1 (Gawlitza et al. 2017; Nassehi et al. 2011; Nassehi et al. 2013). Measurements of PTBE were performed without knowledge of the histological diagnosis and immunohistochemical or PCR findings.

## Immunohistochemistry

Consecutive (semi-serial) 4-μm sections of formalin-fixed, paraffin-embedded tissue samples were studied. One section for each sample was stained with H&E. The histological sections were deparaffinized in xylene and rehydrated in graded alcohols up to water. Antigen retrieval was performed by microwaving the slides in 0.01 M citrate buffer (pH 6). Endogenous peroxidase activity was quenched by treatment with 1% hydrogen peroxide for 20 min. Incubation with an appropriate protein blocking solution was performed. Sections were subsequently incubated with primary antibodies anti-TRPV4 rabbit polyclonal antibody of Abcam (ab39260; Cambridge, UK) (dilution 1:200), anti AQP4 (4/18) mouse monoclonal antibody (sc32739) of Santa Cruz (Heidelberg, Germany) (dilution 1:50), anti AQP4 (4/18) of Abcam (ab9512; Cambridge, UK) (dilution 1:100), anti-CD31 (JC70A) of DAKO (CA, USA). Detection was carried out using the Envision Plus Detection System kit, according to the manufacturer’s instructions (DakoCytomation, USA), with 3,3′-diaminobenzidine (DAB) as a chromogen (which yielded brown reaction products). Sections were counterstained with Mayer’s hematoxylin solution, dehydrated, and mounted. To ensure antibody specificity, negative controls included the omission of primary antibody and substitution with non-immune serum. Positive human inflammatory bowel for TRPV4 (Rizopoulos et al. 2018) and kidney for AQP4 were included (Mobasheri et al. 2007).

## Scoring of Immunohistochemical Staining

To determine the labeling index (LI) (% labeled cells) for each antibody, ten non-overlapping, random fields (×400 total magnification) for each case were examined and 100 tumor cells in each field with the aid of an ocular grid were manually counted. Immunopositive endothelial and stromal cells were excluded from the cell counts. Meningiomas were regarded as immunopositive when more than or equal to 10% of tumor cells were immunoreactive (LIs ≥ 10). Microphotographs were obtained using a Nikon DXM 1200C digital camera mounted on a Nikon Eclipse 80i microscope and ACT-1C software (Nikon Instruments Inc., Melville, NY, USA).

## Determination of Microvessel Density

Microvessel density determination was assessed as previously described (Assimakopoulou et al. 1997). Briefly, light microscopy at ×100 magnification was used to identify regions within the tumor that contained the greatest microvessel density; extratumoral leptomeningeal blood vessels were ignored. Whenever a highly vascularized area was encountered, individual microvessels were counted at ×200 (×20 objective and ×10 ocular lenses; 0.7386 mm^2^ per field) magnification in this area. Neither vessel lumens nor red blood vessels were used to define a microvessel. Microvessel count (MVC) was expressed as the highest number of microvessels in the three areas of highest vascular density at ×200 magnification that were examined.

## Gene Expression Analysis of AQP4 and TRPV4 by Quantitative Real-Time PCR

Total messenger RNA (mRNA) was extracted from 23 patients whose measurement of PTBE was performed using the commercially available kit, NucleoSpin® totalRNA FFPE Kit (Macherey-Nagel, GmbH & Co., Düren, Germany), according to the manufacturer’s instructions. A total of 3 μg of RNA was reverse transcribed according to the Superscript III Reverse Transcriptase protocol (Life Technologies, Carlsbad, CA, USA). The primers used for real-time polymerase chain reaction were AQP4-sense AGCAATTGGATTTTCCGTTG, AQP4-antisense TGAGCTCCACATCAGGACAG, TRPV4-forwTGGGATCTTTCAGCACATCATC, TRPV4-reverse GAGACCACGTTGATGTAGAAGG. Cycling conditions were 95 °C for 10 min, 95 °C for 15 s, 60 °C for 60 s, for 40 cycles followed by 95 °C for 15 s, 60 °C for 60 s, and 95 °C for 15 s. The PCRs were performed in triplicate in an Mx3000P (Stratagene, La Jolla, CA, USA) cycler. LinRegPCR software was used to quantify detected signals.

## Statistical Analysis

Nonparametric methods were used for statistical analysis of the results. Median comparisons were performed with Wilcoxon’s rank-sum test (equivalent to the Mann-Whitney *U* test) and the Kruskal-Wallis test. Correlations between clinicopathologic data and LIs were analyzed with the Spearman correlation coefficient. The relationships between EI and other variables (AQP4, TRPV4, and MVC) were examined with multivariate linear regression analysis. Overall survival (OS) was analyzed using the Kaplan-Meier method, and differences between subgroups were compared with the log-rank-test (the mean values were used as cut-off points to classify tumors exhibiting low and high protein expression or MVC). Cox proportional hazard univariate analysis was performed to identify predictors of survival and the relative risk was calculated with 95% confidence interval (CI). *p* values < 0.05 were considered significant.

## Results

### AQP4/TRPV4 Co-expression in Meningothelial Cells and Tumor Endothelium

**Fig. 1:**
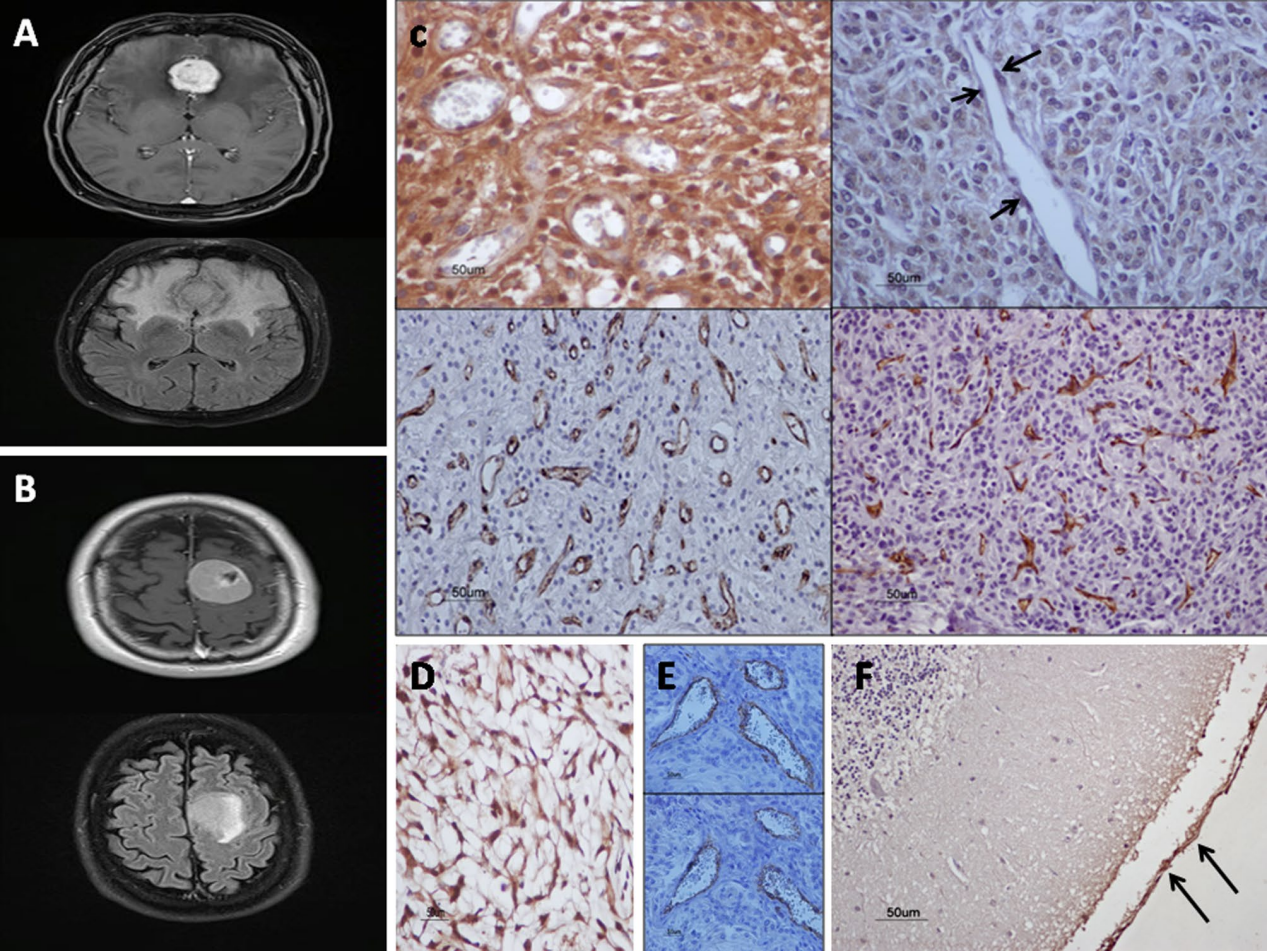
Magnetic resonance imaging and immunohistochemical findings for AQP4 and TRPV4 expression and microvessel density in meningiomas. **a**, **b** Axial sections T1-weighted images with contrast enhancement (upper panels) and T2-weighted fluid-attenuated inversion recovery images (lower panels) depicting tumor and peritumoral edema. **c** Immunohistochemistry for AQP4 with LI = 90 (left upper panel) and high microvascular density (left lower panel) in an edematous patient with angiomatous meningioma (WHO, grade I). The tumor demonstrates also TRPV4 strong immunoexpression (LI = 80) (not shown). The edema index is 3.74. Immunohistochemistry for TRPV4 with LI = 80 (right upper panel) and high microvascular density (right lower panel) in a patient with high-grade meningioma (WHO, grade III). Note the nuclear immunolocalization of TRPV4 in endothelial cells (arrows). AQP4 immunostaining was negative for this tumor. **d** Strong AQP4 immunoreactivity (LI = 95) for a chordoid meningioma (WHO, grade II). **e** Homologous fields from the same meningioma (WHO, grade II) presenting AQP4-immunopositive endothelium of vessels, whereas the tumor cells are AQP4-immunonegative (lower panel), and the vessels highlighted with CD31 (upper panel). **f**, Leptomeninges found in normal brain tissue are AQP4-immunopositive (*arrows*) and TRPV4-immunonegative (not shown). × 400, counterstain, hematoxylin; scale bar 50 μm

Meningothelial cells of meninges, found in normal brain specimens and in some tumors, were weakly or moderately AQP4-immunopositive and TRPV4-immunonegative. Meningioma cells demonstrated moderate to strong cytoplasmic immunoreactivity for AQP4 and TRPV4 channel expression. In 35% of meningiomas, AQP4/TRPV4 co-expression in ≥ 10% tumor cells (LI ≥ 10) was observed over a wide range in the tumor body but its expression in tumoral tissues nearby or to invading the meninges increased significantly. Fifty-eight percent of meningiomas were AQP4 immunonegative (LI < 10) and TRPV4 immunopositive (LI ≥ 10) or AQP4/TRPV4 immunonegative (LI < 10). A few cases (7%) were AQP4 immunopositive (LI ≥ 10) and TRPV4 immunonegative (LI < 10). In some tumors, irrespective of the AQP4 immunoreactivity of tumor cells, endothelial cells displayed AQP4 cytoplasmic immunoreactivity. TRPV4 nuclear immunoreactivity was detected in endothelial cells of high-grade meningiomas (grade II and III). AQP4/TRPV4 co-localization was not detected in endothelium of tumor capillaries (Fig. 1).

### Quantitative Analyses of AQP4/TRPV4 Channel Immunoexpression and MVC

AQP4 expression levels were significantly decreased in grade II and grade III meningiomas compared with grade I meningiomas (*p* = 0.02). Comparison of median LIs showed that TRPV4 expression levels were significantly increased than AQP4 expression, in all grades (Table 1). There was a significant correlation between AQP4 and TRPV4 expression levels in benign (WHO, grade I) (Spearman rho = 0.366, *p* < 0.0001) but not in high-grade (WHO, grades II and III) meningiomas (*p* ≥ 0.05). Multivariate regression analysis showed that AQP4 status would predict TRPV4 status (*p* < 0.0001). AQP4 and TRPV4 did not correlate with patient’s age or gender (*p* ≥ 0.05). AQP4 and TRPV4 expression was independent of MVC and proliferation index of the tumors (*p* ≥ 0.05). Patients with age ≥ 60 years demonstrated significantly increased MVC values compared with patients < 60 years old (*p* = 0.009). MVC values were increased in high-grade (WHO, grades II and III) meningiomas compared with benign (WHO, grade I) meningiomas, and in male compared with female, although the differences were not significant (Table 1).

### EI in Association with AQP4/TRPV4 and MVC

**Fig. 2:**
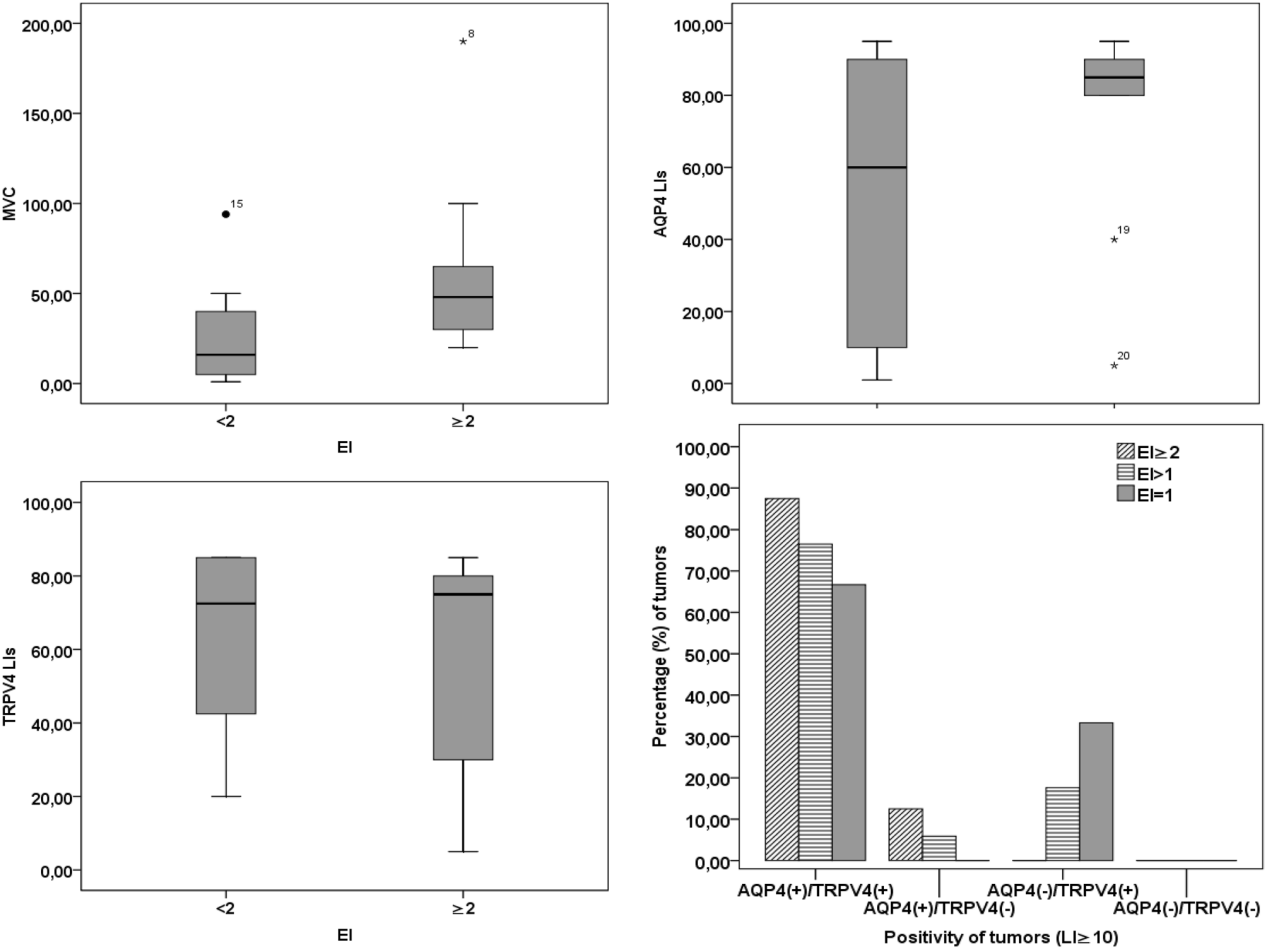
Comparison of EI values in meningiomas according to AQP4 and TRP4 expression and MVC (**a**–**c**) and AQP4/TRPV4 co-expression profiles in edematous and non-edematous meningiomas (**d**)

**Fig. 3:**
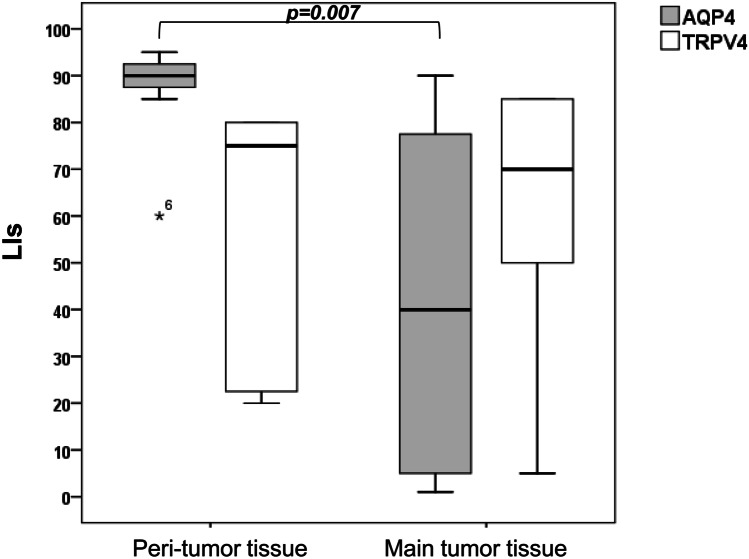
AQP4 and TRPV4 channel expression in peri-tumor (peri-meningioma tissue) compared with main tumor tissue of edematous meningiomas (n = 20). The (non-parametric) Wilcoxon’s rank-sum test was used and the level of significant was defined as p < 0.05

Twenty patients (17, WHO, grades I and 3, WHO, grade II) had edema, while three patients (WHO, grade I) had no edema. Evaluation of AQP4/TRPV4 expression in peri-meningioma (peri-tumor) tissue compared with the main tumor of edematous meningiomas revealed significantly increased expression for AQP4 (*p* = 0.007) but not for TRPV4 (*p* > 0.05) in peri-meningioma tissue (Table 1; Fig. 2). A proportion of edematous (76.5%) and non-edematous (66.7%) meningiomas demonstrated co-expression in ≥ 10% of tumor cells (LI ≥ 10%) of AQP4 and TRPV4 channels. mRNA levels of AQP4 and TRPV4 did not differ between edematous and non-edematous tumors (*p* ≥ 0.05). EI was not associated with AQP4 and/or TRPV4 expression (*p* ≥ 0.05). However, in the majority (87.5%) of tumors with EI ≥ 2, AQP4/TRPV4 co-expression (LI ≥ 10%) was detected (Fig. 3) and AQP4 expression was increased compared with tumors with EI < 2, but the difference was not significant (*p* ≥ 0.5) (Table 1). In contrast, EI was significantly correlated to MVC (Spearman’s rho = 0.485, *p* = 0.02). Comparison of median MVC between tumors with EI ≥ 2 and tumors with EI < 2 revealed significantly increased MVC in tumors with larger edema EI ≥ 2 (*p* = 0.01). No correlation between EI and tumor size or grade was found (*p* ≥ 0.05). EI values did not correlate with patient’s age or gender (*p* > 0.05). However, increased mean EI was found in group of patients ≥ 60 years old compared with the group of patients < 60 years although the difference was not significant (*p* > 0.05).

### AQP4/ TRPV4 Expression, MVC, and EI in Association with Patient Survival

AQP4/TRPV4 levels or MVC (low versus high protein expression or MVC) and EI (EI = 1 vs EI > 1) were not associated with patient survival (log-rank *p* > 0.05).

## Discussion

Previous knowledge provides evidence for the distribution of AQP4 in meningothelial cells (Mobasheri et al. 2007; Zeleny et al. 2017). Since these cells are responsible for CSF drainage into the dural sinuses and veins (Weller et al. 2018), the presence of AQP4 water channels may play a role in solute transport across the meningothelial layer. Previous studies in meningiomas, the neoplastic counterpart of meningothelial cells, proposed AQP4 as a possible factor in PTBE formation in terms of abnormal water transport over cell membranes (Gawlitza et al. 2017; Ng et al. 2009; Wang et al. 2011). In the present study, expression of AQP4 was analyzed in meningioma samples taken from 174 patients, and in leptomeninges found in normal brain specimens and in some tumors. Cytoplasmic AQP4 expression was detected in meningothelial cells of leptomeninges and meningiomas. Quantification of AQP4 expression, using the labeling index (LI), revealed statistically lower levels of AQP4 in high-grade meningiomas (WHO, grades II and III) compared with WHO, grade I meningiomas. Only in one other study AQP4 expression has been evaluated in different grades of meningiomas (Gawlitza et al. 2017) and was found that the cytoplasmic AQP4 expression is not dependent on tumor grade. The different scoring approaches for AQP4 evaluation between the two studies may be a potential explanation for these controversial results. Furthermore, in the present study, in contrast with previous studies (Gawlitza et al. 2017; Ng et al. 2009; Wang et al. 2011), monoclonal antibodies for the detection of AQP4 protein were used. Nevertheless, the higher expression in low-grade cells may indicate a closer to normal meningothelial cell response to edema.

In the group of meningiomas, where PTBE was measured, only three non-edematous meningiomas (WHO, grade I) were found. However, increased expression of AQP4 in edematous vs non-edematous meningiomas as well as in meningiomas with larger edema (EI ≥ 2), was detected, confirming previous findings (Gawlitza et al. 2017; Ng et al. 2009; Wang et al. 2011). This differential expression of AQP4 between the two groups of meningiomas reached no significance because of the small number of patients in the group with non-edematous meningiomas (*n* = 3). Additionally, Gawlitza et al. (2017) found a significant correlation between EIs and AQP4 expression using the parametric Pearson correlation test and linear regression analysis, which was missed with the non-parametric Spearman’s *ρ*. Similarly, in the present study, there was not a significant correlation of AQP4 expression with EIs using Spearman’s correlation test. Until now, the role of AQP4 in PTBE of meningiomas is not clear given that in vasogenic edema, water entry in the brain is AQP4-independent contrary to cytotoxic brain edema which is AQP4-dependent (Papadopoulos et al. 2004b; Papadopoulos and Verkman 2007; Zador et al. 2009). However, intriguing data from models of vasogenic brain edema in mice have shown that AQP4 facilitates reabsorption of excess fluid in vasogenic brain edema (Papadopoulos et al. 2004a). Indeed, AQP4 was significantly overexpressed in peri-meningioma tissue compared with the main tumor of edematous meningiomas.

Moreover, interactions between AQP4 and TRPV4, a gated ion channel with responsiveness to osmolarity, as well as some specific ligands, have been experimentally proved to regulate the cerebral volume control in astrocytes (Benfenati et al. 2011; Iuso and Križaj 2016; Jo et al. 2015) and contribute to CNS edema formation (Kitchen et al. 2020). In the present study, we detected the co-expression of AQP4 and TRPV4 in 35% of meningiomas, specifically grade I. This finding drove us to investigate the relationship of this co-expression with edema formation and edema extent in these tumors. Peritumoral edema was unrelated to the expression of TRPV4 and much more neither the combination of AQP4 and TRPV4 since 76.5% of edematous and 66.7% of non-edematous meningiomas showed co-expression of AQP4 and TRPV4 channels. However, co-expression of AQP4 and TRPV4 was associated with more extensive edema (EI ≥ 2). Thus, as AQP4 may be in fact a feedback response to vasogenic edema, the co-upregulation and presumed synergistic role of AQP4 and TRPV4 in meningiomas with larger edema could also be a response to edema. These are contradicting data compared with a recent evidence for cooperation of TRPV4 with AQP4 in astrocytes which results in edema formation (Kitchen et al. 2020) and may support the theory that PTBE in meningiomas depends on different mechanism from that of cytotoxic edema. Functional experiments would clarify the exact action of a possible cooperation of AQP4 and TRPV4 in pathogenesis of meningiomas. However, recently, AQP4/TRPV4 complex has been shown to be deleterious in therapeutic intervention for edema using hypothermia (Salman et al. 2017).

Finally, we wanted to correlate the above results with neovascularization, which is a well-established factor related to PTBE formation in meningiomas (Hou et al. 2013; Nassehi et al. 2011; Nassehi et al. 2013; Nassehi 2013; Sakuma et al. 2008; Schmid et al. 2010). We used the routinely performed MVC to measure the microvascular densities in meningiomas. Our data suggest that the microvascular density, contrary to AQP4 and/or TRPV4 expression, is strongly associated with PTBE extent in meningiomas. However, the fact that AQP4 and TRPV4 immunoexpression was independent of MVC indicates that different mechanisms from that of angiogenesis may be activated for the expression of these channels. Notably, MVC values were increased in high-grade (WHO, grades II and III) meningiomas compared with benign (WHO, grade I) meningiomas confirming previous data (Ling et al. 2016). WHO, grade II meningiomas have been reported to demonstrate extended PTBE (Ressel et al. 2019), which may depends on high MVC values, but this hypothesis was not investigated in the present study due to the small sample (*n* = 3) of edematous WHO, grade II meningiomas. Additionally, patients with age ≥ 60 years old demonstrated significantly increased MVC values compared with patients < 60 years old, contrary to Barresi et al. (2007) who did not detect a correlation between age and vascularization of meningiomas. These conflicting findings may be explained by the difference in antibodies which were used for the evaluation of microvessel density of the tumors (CD105 vs CD31 in the present study) and patients population studied (54 vs 174 in the present study). Finally, since age was correlated with vascularization and vascularization was correlated with edema, the group of patients with age ≥ 60 years exhibited increased mean EI compared with the group of patients < 60 years old.

In conclusion, in this study, we report high AQP4 expression which was significantly correlated with TRPV4 expression in benign meningiomas. It does not seem that either AQP4 and/or TRPV4 correlate with PTBE formation in contrast to microvessel density which was strongly associated with edema extent. However, increased levels of AQP4 and AQP4/TRPV4 co-expression were detected in most tumors with larger edema whereas upregulation of AQP4 was found in peri-tumor tissue. Given that the formation of PTBE is a multifactorial process, it is challenging to clarify the mechanisms that account for it to identify the patients at risk and provide possible combined therapeutic targets.

## Data Availability

The datasets used and/or analyzed and materials from the current study are available from the corresponding author on reasonable request.
