# Correction to: Evaluation of AQP4/TRPV4 Channel Co-expression, Microvessel Density, and its Association with Peritumoral Brain Edema in Intracranial Meningiomas

**DOI:** 10.1007/s12031-022-01973-4

**Published:** 2022-01-28

**Authors:** Konstantinos Faropoulos, Afroditi Polia, Chrisi Tsakona, Eleanna Pitaraki, Athanasia Moutafidi, George Gatzounis, Martha Assimakopoulou

**Affiliations:** 1 Department of Neurosurgery, University Hospital of Patras, Patras, 26504 Greece; 2 Department of Anatomy, Histology and Embryology, School of Medicine, University of Patras, Patras, 26504 Greece

## Correction to: Journal of Molecular Neuroscience (2021) 71:1786–1795 https://doi.org/10.1007/s12031-021–01801-1

The article “Evaluation of AQP4/TRPV4 Channel Co-expression, Microvessel Density, and its Association with Peritumoral Brain Edema in Intracranial Meningiomas”, written by Konstantinos Faropoulos, Afroditi Polia, Chrisi Tsakona, Eleanna Pitaraki, Athanasia Moutafidi, George Gatzounis, and Martha Assimakopoulou was originally published Online First without Open Access. After publication in volume 71, issue 9, page 1786–1795, the author decided to opt for Open Choice and to make the article an Open Access publication. Therefore, the copyright of the article has been changed to © The Author(s) 2022 and the article is forthwith distributed under the terms of the Creative Commons Attribution 4.0 International License, which permits use, sharing, adaptation, distribution, and reproduction in any medium or format, as long as you give appropriate credit to the original author(s) and the source, provide a link to the Creative Commons licence, and indicate if changes were made. The images or other third-party material in this article are included in the article’s Creative Commons licence, unless indicated otherwise in a credit line to the material. If material is not included in the article’s Creative Commons licence and your intended use is not permitted by statutory regulation or exceeds the permitted use, you will need to obtain permission directly from the copyright holder. To view a copy of this licence, visit http://creativecommons.org/licenses/by/4.0.

The original article has been corrected.

